# Cholinesterase Inhibitors for Lewy Body Disorders: A Meta-Analysis

**DOI:** 10.1093/ijnp/pyv086

**Published:** 2015-07-28

**Authors:** Shinji Matsunaga, Taro Kishi, Ichiro Yasue, Nakao Iwata

**Affiliations:** Department of Psychiatry, Fujita Health University School of Medicine, Toyoake, Aichi, Japan (Drs Matsunaga, Kishi, Yasue, and Iwata).

**Keywords:** cholinesterase inhibitors, Lewy body disorders, Parkinson’s disease, dementia with Lewy bodies, meta-analysis

## Abstract

**Background:**

We performed a meta-analysis of cholinesterase inhibitors for patients with Lewy body disorders, such as Parkinson’s disease, Parkinson’s disease dementia, and dementia with Lewy bodies.

**Methods:**

The meta-analysis included only randomized controlled trials of cholinesterase inhibitors for Lewy body disorders.

**Results:**

Seventeen studies (n = 1798) were assessed. Cholinesterase inhibitors significantly improved cognitive function (standardized mean difference [SMD] = −0.53], behavioral disturbances (SMD = −0.28), activities of daily living (SMD = −0.28), and global function (SMD = −0.52) compared with control treatments. Changes in motor function were not significantly different from control treatments. Furthermore, the cholinesterase inhibitor group had a higher all-cause discontinuation (risk ratio [RR] = 1.48, number needed to harm [NNH] = 14), discontinuation due to adverse events (RR = 1.59, NNH = 20), at least one adverse event (RR = 1.13, NNH = 11), nausea (RR = 2.50, NNH = 13), and tremor (RR = 2.30, NNH = 20).

**Conclusions:**

Cholinesterase inhibitors appear beneficial for the treatment of Lewy body disorders without detrimental effects on motor function. However, a careful monitoring of treatment compliance and side effects is required.

## Introduction

Lewy body disorders, such as Parkinson’s disease (PD), PD dementia (PDD), and dementia with Lewy bodies (DLB), are neurodegenerative diseases characterized by accumulation of Lewy bodies in brain cells (Lippa et al., 2007). Cognitive impairment is an important feature of all Lewy body disorders (Goldman et al., 2014).

A recent meta-analysis showed that cholinesterase inhibitors (ChEIs) (donepezil, galantamine, and rivastigmine) were superior to placebo in improving cognitive function in patients with AD (Tan et al., 2014). In addition, ChEIs have recently been tested for the treatment of Lewy body disorders based on studies reporting cholinergic system dysfunction in these patients (Candy et al., 1983; Tiraboschi et al., 2000; Bohnen et al., 2003). The efficacy of ChEIs in managing patients with DLB, PDD, and cognitive impairment in PD (CIPD) was assessed in a Cochrane meta-analysis of 6 randomized controlled trials (RCTs) including 1263 patients (Rolinski et al., 2012). This meta-analysis revealed that pooled ChEIs were superior to placebo in improving cognitive function in patients with DLB, PDD, and CIPD (standardized mean difference [SMD] = −0.34, 95% confidence interval [CI] = −0.46 to −0.23, *P* < .00001]. Wang et al. (2015) conducted a meta-analysis of 7 RCTs (1403 patients) evaluating ChEIs (donepezil and rivastigmine) and memantine for DLB, PDD, and CIPD; results revealed that donepezil and rivastigmine were superior to placebo in improving cognitive function, as assessed by Mini-Mental State Examination (MMSE) (Folstein et al., 1975) in patients with DLB, PDD, and CIPD (5-mg donepezil: weighted mean difference [WMD] = −2.57, 95% CI = −4.23 to −0.90, *P* = .003, 3 RCTs, n = 440; 10mg donepezil: WMD = −1.31, 95% CI = −2.53 to −0.09, *P* = .04, 4 RCTs, n = 450; and 12-mg rivastigmine: WMD = −1.04, 95% CI = −1.65 to −0.43, *P* = .0009, 2 RCTs, n = 621].

As PD is a Lewy body disorder, we performed a meta-analysis of ChEI safety and efficacy for treating patients with Lewy body disorders, including DLB, PDD, CIPD, and PD. This analysis pooled the results of 17 RCTs (involving 1798 patients) using the same methodology as that used in our previous meta-analysis (Matsunaga et al., 2015).

## Methods

This meta-analysis was performed according to Preferred Reporting Items for Systematic Reviews and Meta-Analysis guidelines (Moher et al., 2010). We systematically reviewed the literature using the PICO strategy (patients: Lewy body disorders; intervention: ChEIs, including donepezil, galantamine, and rivastigmine; comparator: placebo or usual care; outcomes: cognitive function [primary], behavioral disturbances [primary], motor function [primary], global function, activities of daily living, discontinuation rate, and individual adverse effects).

### Inclusion Criteria, Search Strategy, Data Extraction, and Outcome Measures

We included only RCTs of ChEIs for patients with Lewy body disorders. Open-label, nonplacebo-controlled (ie, usual care), and crossover studies were included for increasing the sample size. To identify relevant studies, we searched PubMed, Cochrane Library databases, EMBASE, CINAHL, and PsycINFO citations. There were no language restrictions, and we considered all studies published up to July 14, 2015. We used the following key words: “cholinesterase inhibitor,” “donepezil,” “galantamine,” “rivastigmine,” “Lewy,” “Parkinson disease,” or “Parkinson’s disease.” Additional eligible studies were sought by searching the reference lists of the primary articles and relevant reviews.

Two authors (S.M. and T.K.) scrutinized the patient inclusion and exclusion criteria for the identified studies. When data required for the meta-analysis were missing, the first and/or corresponding authors were contacted for additional information, including endpoint scores. Three authors (S.M., T.K., and I.Y.) independently extracted, assessed, and entered the data into Review Manager (Version 5.3 for Windows, Cochrane Collaboration, http://ims.cochrane.org/revman). Discrepancies in different coding forms were resolved by discussions between authors (S.M. and T.K.)

### Data Synthesis and Statistical Analysis

Each outcome measure reported in this study was used in at least 3 of the 17 included studies. The primary outcome measures of efficacy were cognitive function, behavioral disturbances, and motor function. Cognitive function was assessed by MMSE, modified MMSE (Teng and Chui, 1987), or Montreal Cognitive Assessment (Dalrymple-Alford et al., 2010). Behavioral disturbances were assessed by Neuropsychiatric Inventory (Cummings et al., 1994) and Brief Psychiatric Rating Scale (Overall and Gorham, 1962). Motor function was assessed by Unified Parkinson’s Disease Rating Scale-motor (UPDRS-motor) (Fahn et al., 1987). Secondary outcome measures included ADL, global function, all-cause discontinuation, discontinuation due to adverse events, and incidence of individual adverse events. ADL was assessed by Alzheimer’s Disease Co-operative Study-Activities of Daily Living Inventory (Galasko et al., 1997), Unified Parkinson’s Disease Rating Scale-Activities of Daily Living (Fahn et al., 1987), and Zarit Caregiver Burden Interview (Zarit et al., 1980). Global function was assessed by Clinician’s Interview-Based Impression of Change plus Caregiver Input (Olin et al., 1996) and Alzheimer’s Disease Cooperative Study-Clinical Global Impression of Change (Schneider et al., 1997).

We based our analyses on intent-to-treat (ITT) or modified ITT data (ie, at least 1 dose or at least 1 follow-up assessment). However, we analyzed the complete set of data for ensuring that the maximum possible information was included (Okereke et al., 2004; Litvinenko et al., 2008).

For combining studies, we used the random effects model of DerSimonian and Laird (1986). This model is more conservative than the fixed effects model and provides a wider CI. For continuous data, we calculated Hedges’ g SMD effect sizes and used the cut-off values for small, medium, and large effect sizes (0.2, 0.5, and 0.8, respectively) set by Cohen (1988). If SD was not reported, it was calculated from the 95% CI (Higgins and Green, 2011). Furthermore, for dichotomous data, the risk ratio (RR) was estimated along with 95% CIs. When the random effects model revealed significant differences between groups, the number needed to harm (NNH) was calculated from the risk difference as NNH = 1/ risk difference. We explored study heterogeneity using the *I* ^*2*^ statistic, with values of ≥50% regarded as considerable heterogeneity (Higgins et al., 2003). In cases with *I* ^*2*^ ≥ 50% for the primary outcome measures, we conducted sensitivity analyses for determining the reasons for heterogeneity. We examined the following confounding factors: blinding (double-blind or not), placebo-controlled or non-placebo–controlled trial, diagnosis (DLB, PDD + CIPD, or PD), trial duration (>12 weeks or ≤12 weeks, as 12 weeks was the median of the study duration for all included trials), type of ChEI (donepezil, galantamine, or rivastigmine), sample size (>28 per group or ≤28 as 28 was the median number of participants per group), sponsorship (industry sponsored or not), and quality of the trial design (high quality trial or “other,” where high quality is defined as double-blind, parallel, and placebo-controlled as well as ITT or modified ITT analysis). Funnel plots were visually inspected for assessing the possibility of publication bias. We also assessed the methodological qualities of the articles included on the basis of the Cochrane risk of bias criteria (Cochrane Collaboration; http://www.cochrane.org/).

## Results

### Characteristics of the Included Studies

**Figure 1.**
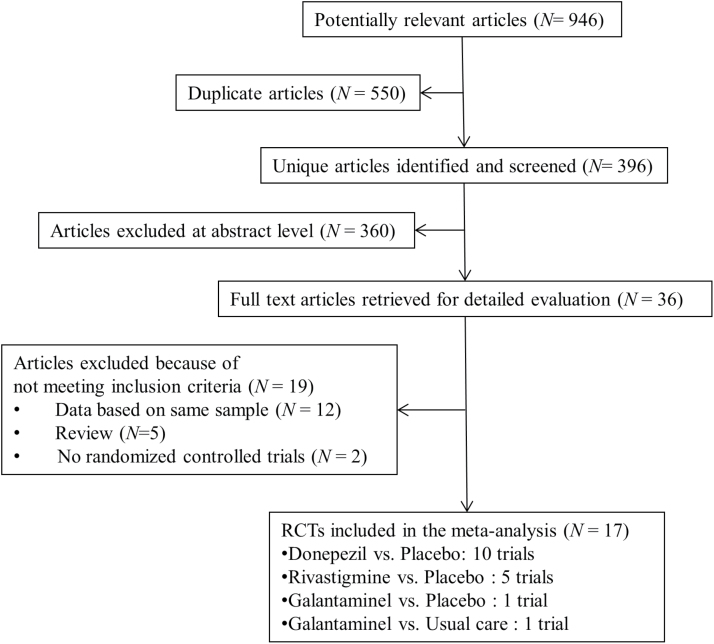
The Preferred Reporting Items for Systematic reviews and Meta-Analysis (PRISMA) flow diagram.

The search yielded a total of 946 references, of which 550 were duplicates (Figure 1). We excluded 360 references for not meeting our criteria after reviewing the title and abstract, and a further 19 were excluded after full-text reviews because they provided duplicate studies (n = 12), review articles (n = 5), or non-RCTs (n = 2). Seventeen RCTs were finally included for testing ChEIs for Lewy body disorders.

**Figure 2.**
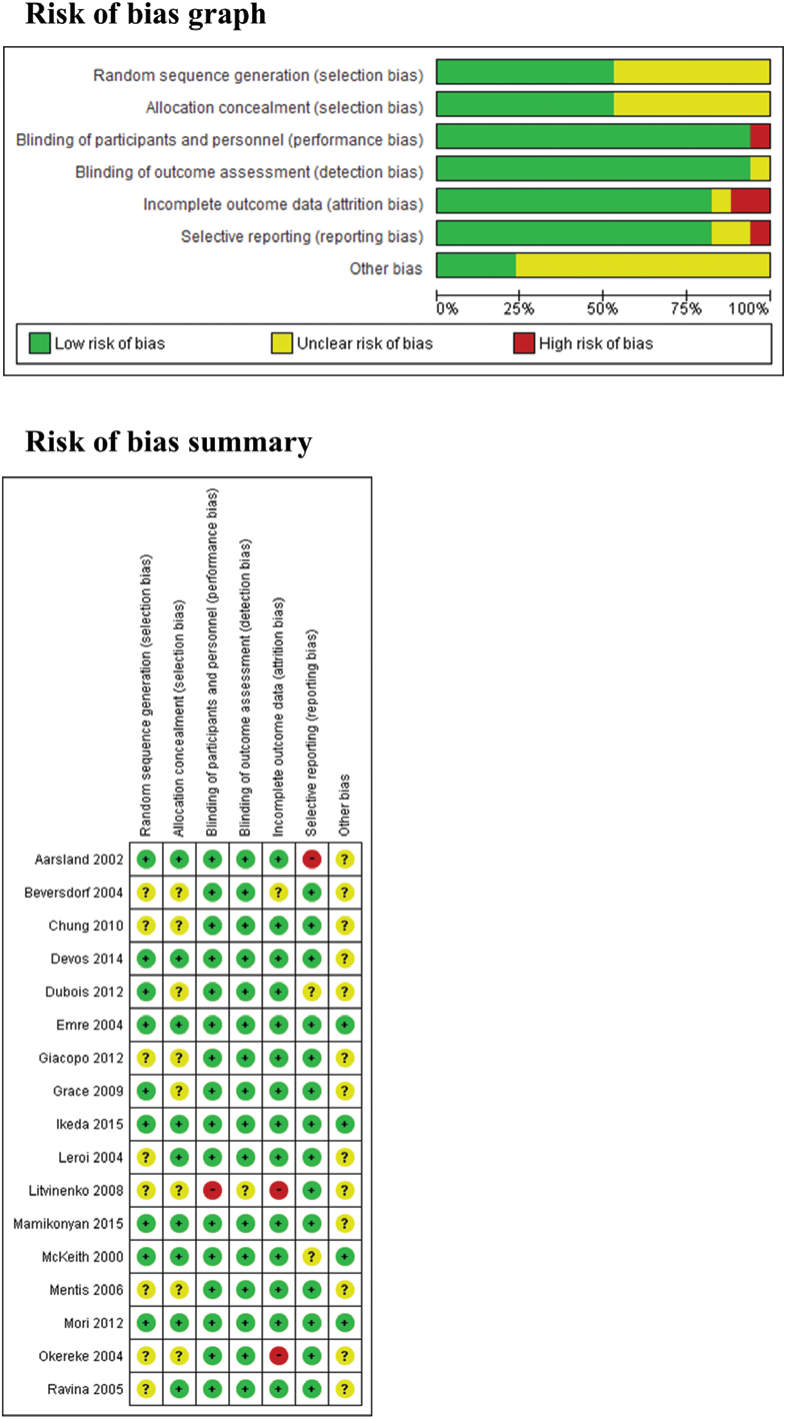
Risk of bias assessment.

The 17 RCTs included 1798 patients (McKeith et al., 2000; Aarsland et al., 2002; Beversdorf et al., 2004; Emre et al., 2004; Leroi et al., 2004; Okereke et al., 2004; Ravina et al., 2005; Mentis et al., 2006; Litvinenko et al., 2008; Grace et al., 2009; Chung et al., 2010; Di Giacopo et al., 2012; Dubois et al., 2012; Mori et al., 2012; Devos et al., 2014; Ikeda et al., 2015; Mamikonyan et al., 2015). The studies included 4 on ChEIs for DLB (McKeith et al., 2000; Beversdorf et al., 2004; Mori et al., 2012; Ikeda et al., 2015), 7 on ChEIs for PDD and CIPD (Aarsland et al., 2002; Emre et al., 2004; Leroi et al., 2004; Ravina et al., 2005; Litvinenko et al., 2008; Dubois et al., 2012; Mamikonyan et al., 2015), and 6 on ChEIs for PD (Okereke et al., 2004; Mentis et al., 2006; Grace et al., 2009; Chung et al., 2010; Di Giacopo et al., 2012; Devos et al., 2014). Ten studies were double-blind, parallel, and placebo-controlled (McKeith et al., 2000; Emre et al., 2004; Leroi et al., 2004; Mentis et al., 2006; Grace et al., 2009; Di Giacopo et al., 2012; Dubois et al., 2012; Mori et al., 2012; Devos et al., 2014; Ikeda et al., 2015), 1 was open-label, parallel, and non-placebo–controlled (Litvinenko et al., 2008), 5 were double-blind, placebo-controlled, and single-crossover (Aarsland et al., 2002; Okereke et al., 2004; Ravina et al., 2005; Chung et al., 2010; Mamikonyan et al., 2015), and 1 was double-blind, placebo-controlled, double-crossover (Beversdorf et al., 2004). The mean study duration was 13 weeks (4–26 weeks). Further, 7 to 550 patients were included in each study. The mean age of the entire study population was 72 years. Twelve of 17 studies were sponsored by pharmaceutical companies. Eight studies were conducted in the United States; 2 in Japan; 1 each in France, Italy, Norway, and Russia; and 3 in multiple countries. The characteristics of the trials included are summarized in Table 1. We evaluated the methodological quality of all studies using the Cochrane risk of bias criteria (Figure 2). Eight studies (Beversdorf et al., 2004; Leroi et al., 2004; Okereke et al., 2004; Ravina et al., 2005; Mentis et al., 2006; Litvinenko et al., 2008; Chung et al., 2010; Di Giacopo et al., 2012) did not mention the method of randomization. Furthermore, 8 studies (Beversdorf et al., 2004; Okereke et al., 2004; Mentis et al., 2006; Litvinenko et al., 2008; Grace et al., 2009; Chung et al., 2010; Di Giacopo et al., 2012; Dubois et al., 2012) did not mention the method of allocation concealment. One study was an open trial (Litvinenko et al., 2008), and 2 (Okereke et al., 2004; Litvinenko et al., 2008) used a complete analysis. One study (Beversdorf et al., 2004) did not report detailed information regarding method of statistical analysis; therefore, we did not include any data in the meta-analysis.

**Table 1.** Characteristics of Included Trials

**Study**	**Total n**	**Patients**	**Diagnosis**	**Duration**	**Age (mean ± SD**)	**Male (%**)	**Race (%**)	**Drug**	**n**	**Dose (dose mg/day**)	**Outcomes**
Aarsland 2002 (Norway) Industry	14	Patients: CIPD Inclusions: H-Y 4 ≧, age 45– 95 years, MMSE 16–26 Exclusions: using anticholinergic drugs or psychotropic drugs with anticholinergic effects	Definite or probable PD: published criteria (Larsen 1994) dementia: DSM-IV	10 weeks (crossover design)	71±3.9	92	NR	DON	12	10mg (flexible dose)	DON > PLA: MMSE, CIBIC-plus DON = PLA: UPDRS-motor, NPI
								PLA	12		
Beversdorf 2004 (USA) Industry	7	Patients: DLB Inclusions: NR Exclusions: NR	DLB: consensus diagnostic criteria	4 weeks (double crossover design)	65±3.47	42.9	NR	DON PLA	7	5 mg	DON > PLA: MMSE, ADAS-cog DON = PLA: BNT, HVLT, VSB, PSMS, IADL, UPDRS
									7		
Chung 2010 (USA) Industry	23	Patients: PD Inclusions: a baseline frequency of falling or nearly falling 2 or more times per week Exclusions: H-Y = V, MMSE < 25, using ChEI or anticholinergic or sedative-hypnotic properties	Probable idiopathic PD: clinical diagnosis	6 weeks (crossover design)	68.3±10.8	65.2	NR	DON	19	10mg. (flexible dose)	DON > PLA: fall frequency (falls/day) DON = PLA: near-fall frequency (near falls/day), CGI-I, ABC, BB, UPDRS-motor, MMSE
								PLA	19		
Devos 2014 (France) Non-industry	30	Patients: PD Inclusions: LARS ≧ 16 Exclusions: dementia (MDS criteria), axis I psychiatric disorders (DSM-IVTR), MADRAS > 18, DBS for less than 2 years, using cholinomimetic drugs or carbamate derivatives, age ≧ 80	PD: Gibb’s criteria	6 months	RIV: 68 PLA: 65 (median)	RIV: 69 PLA: 57	NR	RIV patch	16	9.5mg (flexible dose)	RIV > PLA: LARS, ZBI, IADL RIV = PLA: PDQ-39, MDRS, UPDRS-motor
								PLA	14		
Dubois 2012 (Germany, Austria, Spain, Russia, UK, France, Australia, New Zealand, South Africa, Canada, Italy, Belgium, Portugal) Industry	550	Patients: PDD Inclusions: age ≧ 40 years, H-Y II-IV, MMSE 10–26 Exclusions: DLB (use consensus diagnostic criteria), previously treated with centrally ChEI, hypersensitivity PIP, using anticholinergics and cholinergic agents	PD: QSBBC dementia: DSM-IV	24 weeks	DON 10 mg: 70.8±7.46 DON 5 mg: 72.0±6.83 PLA: 72.9±6.48	DON 10 mg: 75 DON 5 mg: 65 PLA: 65	DON 10 mg: White 98 Black 1 Other 0.5 DON 5 mg: White 99 Black 0 Other 1 PLA: White 100 Black 0 Other 0	DON 10 mg	182	10mg (fixed dose)	DON 10mg > PLA: CIBIC-plus, MMSE, VF (category), VF (letter), VF (switching), BTA DON 10mg = PLA: ADAS-cog, DAD, NPI, SE DON 5mg > PLA: MMSE, VF (category), VF (letter), VF (switching), BTA DON 5mg = PLA: ADAS-cog, CIBIC-plus, DAD, NPI, SE
								DON 5 mg	195	5mg (fixed dose)	
								PLA	173	PLA	
Emre 2004 (Austria, Belgium, Canada, France, Germany, Italy, Netherlands, Norway, Portugal, Spain, Turkey, and UK) Industry	541	Patients: PDD Inclusions: age ≧ 50 years, MMSE 10–24, the onset of symptoms occurring at least two years after the diagnosis of PD Exclusions: hypersensitivity RIV or similar drugs, using ChEI or anticholinergic drugs	PD: UKPDSBB dementia: DSM-IV	24 weeks	RIV: 72.8±6.7 PLA: 72.4±6.4	RIV: 64.6 PLA: 65.4	RIV: White 99.4 Other 0.6 PLA: White 100 Other 0	RIV	362	12mg (flexible dose)	RIV > PLA: ADAS-cog, ADCS-CGIC, ADCS-ADL, NPI-10, MMSE, CDRCAS, VF, CDT RIV = PLA: UPDRS-motor
								PLA	179		
Giacopo 2012 (Italy) Non-industry	12	Patients: PD Inclusions: RBD, RBD phenomenon refractory to MEL and CLO Exclusions: dementia, using anticholinergics or antidepressants and DBS	PD: NR	3 weeks (crossover design)	67.7±7.3	91.7	NR	RIV patch	10	4.6mg (fixed dose)	RIV > PLA: RBD episode frequency
								PLA	10		
Grace 2009 (USA) Non-industry	69	Patients: PD Inclusions: age 40–90 years, at least 6 years of formal education Exclusions: dementia (DSM- IV), 3MS < 77, GDS > 7, using antipsychotic or anticholinergic medication	PD: UKPDSBB	16 weeks	GAL: 65.9±9.6 PLA: 68.8±10.0	GAL: 78.9 PLA: 67.7	NR	GLA	38	24mg (fixed dose)	GLA = PLA: CPT, COWA, 3MS, HVLT, TMT-A, TMT-B, OSDM, CDT, CWT, Clarity of thinking (VAS), PDQ-39, FrSBE, CB, NPI-Q, UPDRS-motor
								PLA	31		
Ikeda 2015 (Japan) Industry	142	Patients: DLB Inclusions: age ≧ 50 years, MMSE 10–26, CDR ≧ 0.5, NPI-plus ≧ 8 and NPI-2 ≧ 1 Exclusions: hypersensitivity to DON or PIP derivatives, H-Y ≧ IV, treatment with ChEIs or any investigational drug within 3 months prior to screening	probable DLB: the consensus diagnostic criteria	12 weeks	DON 10 mg: 77.7±6.8 DON 5 mg: 78.8±5.1 PLA: 77.2±6.1	DON 10 mg: 42.9 DON 5 mg: 44.4 PLA: 38.6	Japanese 100	DON 10 mg	49	10mg (fixed dose)	DON 10mg > PLA: MMSE DON 10mg = PLA: NPI-10, NPI-2, ZBI, UPDRS-motor DON 5mg = PLA: MMSE, NPI-10, NPI-2, ZBI, UPDRS-motor
								DON 5 mg	47	5mg (fixed dose)	
								PLA	46		
Leroi 2004 (USA) Industry	16	Patients: PDD or CIPD Inclusions: on stable regimens of antiparkinsonian medication Exclusions: MMSE < 10, known inability to tolerate DON	PD: UKPDSBB dementia or cognitive impairment: DSM-IV	18 weeks	DON: 66.2±9.3 PLA: 70.8±11.8	DON: 85.7 PLA: 44.4	NR	DON	7	10mg (flexible dose)	DON > PLA: MDRS memory subscore DON = PLA: MMSE, MDRS, MDRS attention, MDRS initiation-perseveration, MDRS conceptual planning, MDRS construction, BTA, TMT-A, TMT-B, VF (sum of FAS), VF (category), HVLT-R total, HVLT-R recall, HVLT-R recognition, VMI, NPI, CSDD, UPDRS-ADL, UPDRS-motor, UPDRS-complication of treatment, H-Y
								PLA	9		
Litvinenko 2008 (Russia) Non-industry	41	Patients: PDD Inclusions: MMSE < 25, presence of dementia developing two years from onset of PD Exclusions: using ChEI or nootropes, HAM-D > 18	PD: UKPDSBB dementia: ICD-10	24 weeks	GAL: 68.6±9.3 UC: 72.6±8.6	NR	NR	GAL	21	16 mg	GLA > PLA: MMSE, ADAS-cog, FAB, CDT, DAD GLA = PLA: UPDRS-motor
								UC	20		
Mamikonyan 2015 (USA) Industry	28	Patients: CIPD Inclusions: age 40–85, CDR = 0.5, DRS-2 < 8, Exclusions: PDD and DLB,	PD: NR MCI: Winblad criteria	10 weeks (crossover design)	64.3±8.2	78.6	White: 96.4	RIV patch	27	9.5mg (flexible dose)	RIV > PLA: ECB RIV = PLA: ADCS-CGIC, MoCa, DRS-2, NCTS, GDS attention, State anxiety subscale of STAI, UPDRS-motor, GDS-15, PPRS, PDQ-8, PDAQ
								PLA	27		
McKeith 2000 (Spain, UK and Italy) Industry	120	Patients: DLB Inclusions: MMSE ≧ 9 Exclusions: H-Y > 3, UPDRS subscore > 3, using neuroleptics, anticholinergics, SEL, or similar drugs	probable DLB: clinical diagnosis	20 weeks	RIV: 73.9±6.5 PLA: 73.9±6.4	RIV: 52.5 PLA: 60.7	NR	RIV	59	12mg (flexible dose)	RIV > PLA: NPI-4, NPI-10. RIV = PLA: CGC-plus, MMSE
								PLA	61		
Study	Total n	Patients	Diagnosis	Duration	Age (mean+/-SD)	Male, %	Race (%)	Drug	n	Dose (dose mg/day)	Outcomes
Mentis 2006 (USA) Industry	18	Patients: PD Inclusions: MMSE >27, improve UPDRS 20% < on motor medication Exclusions: taking anticholinergics, dementia	PD: NR	8 weeks	NR	DON: 80 PLA: 25	NR	DON	11	10mg (fixed dose)	DON = LA: Outward movement, Out-and-back movement, Onset time, Timing error, Directional error.
								PLA	7		
Mori 2012 (Japan) Industry	140	Patients: DLB Inclusions: age ≧ 50 years, MMSE 10–26, CDR ≧ 0.5, NPI-plus ≧ 8 Exclusions: H-Y ≧ IV, treatment with ChEI or any investigational drug within 3 months prior to screening	probable DLB: the consensus diagnostic criteria	12 weeks	DON 10 mg: 78.6±6.1 DON 5 mg: 77.9±6.8 DON 3 mg: 79.6±4.5 PLA: 78.6±4.7	DON 10 mg: 11.1 DON 5 mg: 50.0 DON 3 mg: 48.6 PLA: 28.1	Japanese 100	DON 10 mg	37	10mg (fixed dose)	DON 10mg > PLA: MMSE, WMS-R a/c, WAIS-III (symbol digit modalities subscale), NPI-10, NPI-4, NPI-2, ZBI, CIBIC-plus DON 10mg = PLA: VF (category), VF (letter), VPTA (form recognition), UPDRS-motor DON 5mg > PLA: MMSE, WMS-R a/c, VF (category), VF (letter), WAIS-III (symbol digit modalities subscale), NPI-10, NPI-4, NPI-2, CIBIC-plus DON 5mg = PLA: VPTA (form recognition), ZBI, UPDRS-motor DON 3mg > PLA: MMSE, WAIS-III (symbol digit modalities subscale), NPI-2, CIBIC-plus DON 3mg = PLA: WMS-R a/c, VF (category), VF (letter), VPTA (form recognition), NPI-10, NPI-4, ZBI, UPDRS-motor
								DON 5 mg	33	5mg (fixed dose)	
								DON 3 mg	35	3mg (fixed dose)	
								PLA	35	PLA	
Okereke 2004 (USA) Industry	25	Patients: PD Inclusions: taking stable doses of L-dopa, patients were to weigh within 20% of their ideal weight Exclusions: use prohibited agent	PD: NR	15 days (crossover design)	74.0±1.9	NR	NR	DON	23	5mg (fixed dose)	DON = PLA: UPDRS-motor
								PLA	23		
Ravina 2005 (USA) Non-industry	22	Patients: PDD Inclusions: age ≧ 40 years, MMSE 17–26 Exclusions: DLB, using cholinergic or anticholinergic agents except AMA or TOL within the 2 weeks prior to screening	PD: clinical diagnosis dementia: DSM-IV	10 weeks (crossover design)	DON/PLA: 75.0±9.8 PLA/DON: 72.1±8.1	DON/PLA: 100 PLA/DON: 60	NR	DON	19	10mg (flexible dose)	DON > PLA: MMSE, CGI-C DON = PLA: ADAS-cog, MDRS, BPRS, UPDRS total, UPDRS-motor
								PLA	19		

ABC: Activities of Balance Confidence, ADAS-cog: Alzheimer’s Disease Assessment Scale cognitive subscale, ADCS-ADL: Alzheimer’s Disease Cooperative Study–Activities of Daily Living, ADCS-CGIC: Alzheimer’s Disease Cooperative Study–Clinician’s Global Impression of Change, ADL: activities of daily living, AMA: amantadine, BB: Berg Balance, BNT: Boston Naming Test, BPRS: Brief Psychosis Rating Scale, BTA: Brief test of Attention, CB: informant-based Cornell-Brown scale for quality of life in dementia, CDR: Clinical Dementia Rating, CDRCAS: Cognitive Drug Research Computerized Assessment System, CDT: clock drawing test, CGC-plus: Clinical Global Change-plus, CGI-C: Clinical Global Impression of change, CGI-I: Clinical Global Impression - Improvement scale, ChEI: cholinesterase inhibitor, CIBIC-plus: Clinician’s Interview-Based Impression of Change Plus Caregiver Input, CIPD: cognitive impairment in Parkinson’s disease, CLO: clonazepam, COWA: Controlled Oral Word Association, CPT: Conners Continuous Performance Test, CSDD: Cornell Scale for Depression in Dementia, CT: computed tomography, CWT: Color-Word Test, DAD: Disability Assessment for Dementia, DBS: deep brain stimulation, DLB: Dementia with Lewy bodies, DON: donepezil, DRS-2: Dementia Rating Scale-2, DSM-IV (TR): Diagnostic and Statistical Manual of Mental Disorders-4th edition (Text Revision), ECB: Everyday Cognition Battery, FAB: Frontal Assessment Battery, FrSBe: Frontal Systems Behavior Scale, GAL: galantamine, GDS: Gordon Diagnostic System, GDS-SF: Geriatric Depression Scale-Short Form, GDS-15: Geriatric Depression Scale-15, HAM-D: Hamilton Rating Scale for Depression, HVLT(-R): Hopkins Verbal Learning Test(-Revised), H-Y: Hoehn and Yahr scale, IADL: Instrumental Activities of Daily Living, ICD: International Classification of Diseases, LARS: the sensitive Lille Apathy Rating Scale, MADRS: Montgomery–Asberg Depression Rating Scale, MDS: the Movement Disorders Society, MDRS: Mattis Dementia Rating Scale, MEL: melatonin, MMSE: Mini-Mental State Examination, MoCa: Montreal Cognitive Assessment, NCTS: Neurotrax Comprehensive Testing Suite, NPI: neuro-psychiatric inventory, NR: Not report, OSDM: Symbol Digit Modalitied Test, PD: Parkinson’s disease, PDAQ: Penn Daily Activities Questionnaire, PDD: Parkinson’s disease dementia, PDQ-8: Parkinson’s Disease Questionnaire-8, PDQ-39: Parkinson’s Disease Questionnaire-39, PPRS: Parkinson Psychosis Rating Scale, PIP: piperidine, PLA: placebo, PSMS: Physical Self-Maintenance Scale, QSBBC: Queen Square Brain Bank Criteria, RBD: rapid eye movement behavior disorder, RIV: rivastigmine, SD: standard deviation, SE: Schwab and England, SEL: selegiline, STAI: State Trait Anxiety Inventory, TMT: Trail Making Test, TOL: tolterodine, UC: usual care, UKPDSBB: the UK Parkinson’s Disease Society Brain Bank clinical diagnostic criteria, UPDRS: unified Parkinson’s disease rating scale, VAS: Visual Analogue Scale, VF: Verbal Fluency, VMI: develop-mental test of Visual-Motor Integration, VPTA: Visual Perception Test for Agnosia, VSB: Verbal Learning Test, WAIS-Ⅲ: Wechsler Adult Intelligence Scale, WMS-R a/c: Wechsler Memory Scale-Revised attention/concentration subscale, ZBI: Zarit Burden Interview, 3MS: Modified Mini-Mental Status Exam

### Meta-Analysis for Primary Outcomes

#### Cognitive Function

**Figure 3.**
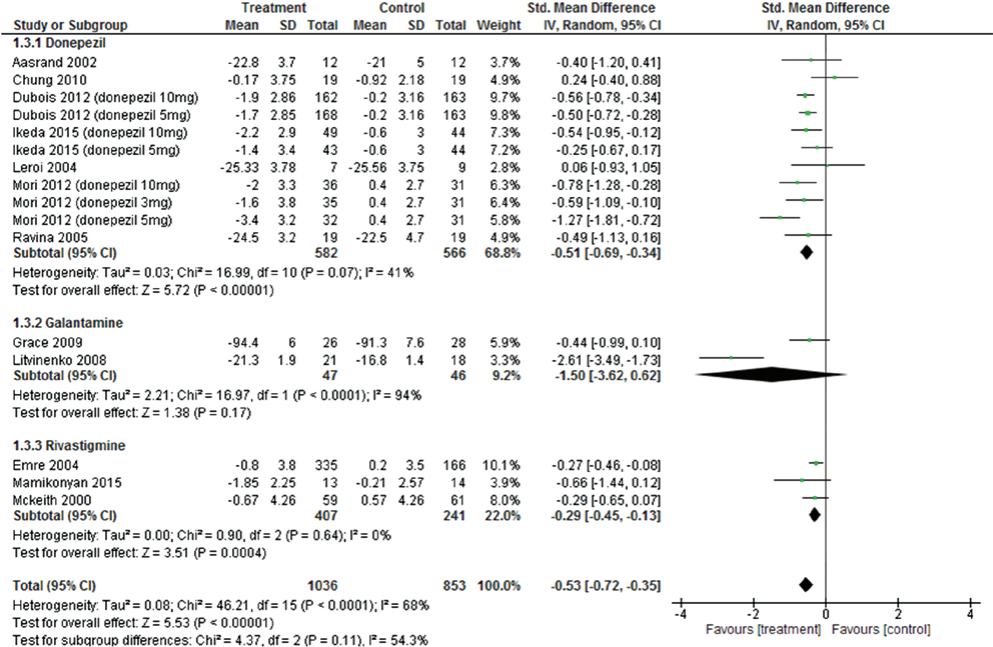
Forest plot of cognitive function (16 comparisons, n = 1889).

**Figure 4.**
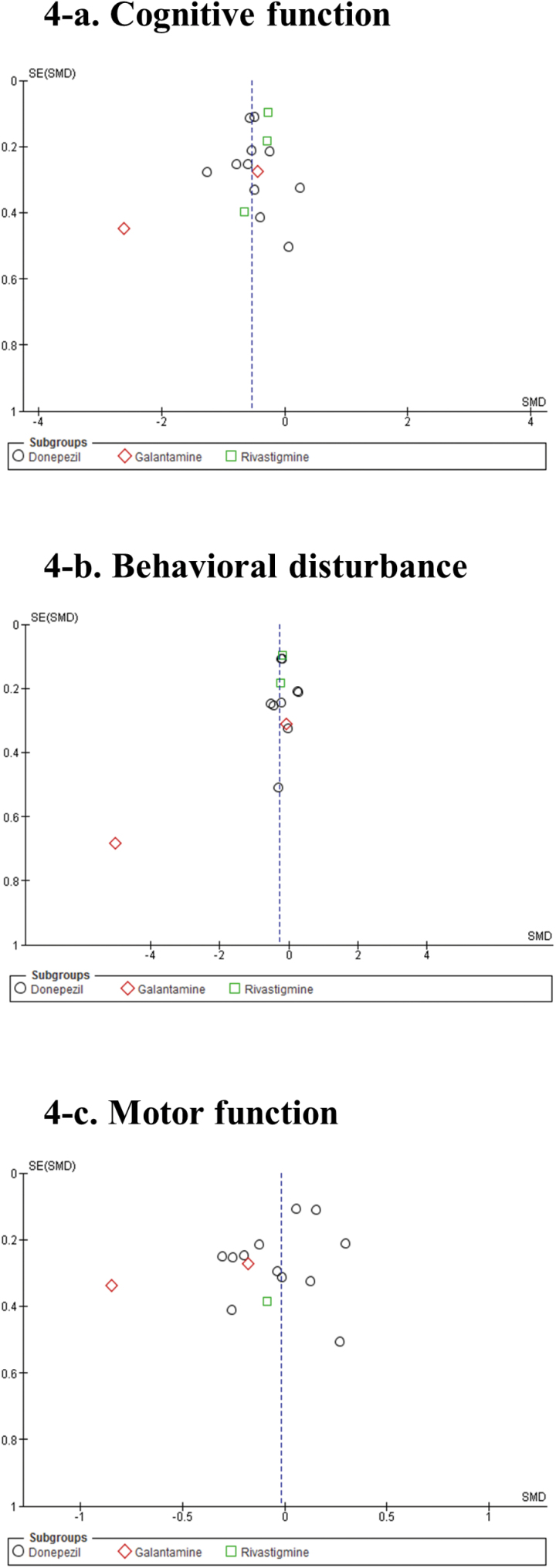
Funnel plots. (a) Cognitive function. (b) Behavioral disturbance. (c) Motor function.

Pooled ChEIs improved cognitive function scores compared with control treatments (SMD = −0.53, 95% CI = −0.72 to −0.35, *P*<.0001, *I* ^2^=68%, 16 comparisons, n=1889) (Figure 3). Visual inspection of the funnel plots for primary outcomes did not suggest the presence of publication bias (Figure 4a). For individual ChEIs, donepezil and rivastigmine significantly improved cognitive function scores compared with placebo (donepezil: SMD=−0.51, 95% CI=−0.69 to −0.34, *P*<.00001, *I* ^2^=41%, 11 comparisons, n=1148 and rivastigmine: SMD=−0.29, 95% CI=−0.45 to −0.13, *P*=.0004, *I* ^2^=0%, 3 comparisons, n=648). In contrast, there was no significant difference in cognitive function scores between galantamine and control groups (SMD=−1.5, 95% CI=−3.62 to 0.62, *P*=.17, *I* ^2^=94%, 2 comparisons, n=93).

#### Sensitivity Analyses of Cognitive Function

There was significant heterogeneity in cognitive function scores among the studies (*I* ^*2*^=68%) (Figure 3). Therefore, we performed several sensitivity analyses for identifying confounding factors affecting cognitive function scores (Table 2a). When divided into a double-blind RCT subgroup and “other” subgroup, the significant heterogeneity disappeared in the double-blind RCT subgroup (double-blind RCTs subgroup [n=15], *I* ^2^=39%; other subgroup [n=1 galantamine study (Litvinenko et al., 2008)], *I* ^2^=not applicable (NA); test for subgroup differences, *I* ^2^=95.6%, *P<*.00001]. When divided into placebo-controlled and non-placebo–controlled trial subgroups, the same results were found. When divided according to the tested ChEIs, the significant heterogeneity disappeared in donepezil and rivastigmine subgroups (donepezil subgroup: *I* ^2^=41%; rivastigmine subgroup: *I* ^2^=0%); however, heterogeneity remained in the galantamine subgroup (*I* ^2^=94%). When divided into DLB, PDD + CIPD, and PD subgroups, there was significant heterogeneity among all subgroups (DLB: *I* ^2^=56%; PDD+CIPD: *I* ^2^=76%; PD: *I* ^2^=61%). ChEIs significantly affected cognitive function scores for DLB and PDD+CIPD subgroups (DLB: SMD=−0.58, 95% CI=−0.86 to −0.31, *P<*.0001, *I* ^2^=56%, 6 comparisons, n=496 and PDD+CIPD: SMD=−0.59; 95% CI=−0.88 to −0.30, *P<*.0001, *I* ^2^=76%, 8 comparisons, n=1301). In contrast, there was no effect of ChEIs on cognitive function scores in the PD subgroup (SMD=−0.12, 95% CI=−0.79 to 0.54, *P*=.72, *I* ^2^=61%, 2 comparisons, n=92). When divided into long duration (>12 weeks) and short duration (≤12 weeks) subgroups, there was significant heterogeneity in both subgroups (long duration subgroup: *I* ^2^=80%; short duration subgroup: *I* ^2^=55%). When divided into high-quality trial (double-blind, randomized, parallel, placebo-controlled trial as well as ITT or modified ITT analysis) and “other” subgroups, the significant heterogeneity disappeared in the high-quality trials subgroup (*I* ^2^=45%) but remained in the “other” subgroup (*I* ^2^=85). Further, when divided into large and small sample size subgroups, the significant heterogeneity disappeared in the small sample size subgroup (*I* ^2^=7%) but remained in the large sample size subgroup (*I* ^2^=75%). When divided into industry and nonindustry subgroups, the significant heterogeneity disappeared in the industry subgroup (*I* ^2^=48%) but remained in the nonindustry subgroup (*I* ^2^=89%).

**Table 2a.** Sensitivity Analysis of Efficacy of Cholinesterase Inhibitors (Cognitive Function)

Variable	Subgroup	N	n	I^2^	SMD	95% CI	P value	Test for Subgroup Differences
Blinding	Double blind	15	1850	39	-0.46	-0.60 to -0.32	**< 0.00001**	I^2^ = 95.6 %, **P < 0.00001**
	Others	1	39	NA	-2.61	-3.49 to -1.73	**< 0.00001**	
Cholinesterase inhibitor	Donepezil	11	1148	41	-0.51	-0.69 to -0.34	**< 0.00001**	I^2^ = 54.3 %, P = 0.11
	Galantamine	2	93	94	-1.5	-3.62 to 0.62	0.17	
	Rivastigmine	3	648	0	-0.29	-0.45 to -0.13	**0.0004**	
Control	Placebo	15	1850	39	-0.46	-0.60 to -0.32	**< 0.00001**	I^2^ = 95.6 %, **P < 0.00001**
	Non-placebo	1	39	NA	-2.61	-3.49 to -1.73	**< 0.00001**	
Diagnosis	DLB	6	496	56	-0.58	-0.86 to -0.31	**< 0.0001**	I^2^ = 0 %, P = 0.43
	PDD + CIPD	8	1301	76	-0.59	-0.88 to -0.30	**< 0.0001**	
	PD	2	92	61	-0.12	-0.79 to 0.54	0.72	
Duration	<12 weeks	8	1413	80	-0.55	-0.82 to -0.28	**< 0.0001**	I^2^ = 0 %, P = 0.91
	≥12 weeks	8	476	55	-0.53	-0.81 to -0.24	**0.0003**	
Quality of the trial design*	High-quality trials trial design	11	1723	45	-0.48	-0.63 to -0.34	**< 0.00001**	I^2^ = 0 %, P = 0.55
	Others	5	166	85	-0.75	-1.62 to 0.11	0.09	
Sample size	Total n > 28	11	1744	75	-0.61	-0.83 to -0.39	**< 0.00001**	I^2^ = 67.2 %, P = 0.08
	Total n ≤ 28	5	143	7	-0.25	-0.59 to 0.10	0.17	
Sponsorship	Industry	13	1758	48	-0.46	-0.62 to -0.31	**< 0.00001**	I^2^ = 16.9%, P = 0.27
	Non-industry	3	131	89	-1.14	-2.33 to 0.06	0.06	

CI, confidence interval; CIPD, cognitive impairment in Parkinson’s disease; DLB, Dementia with Lewy bodies; NA, not applicable; PD, Parkinson’s disease; PDD, Parkinson’s disease dementia; SMD, standardized mean difference.

*High-quality trials trial design: double-blind, parallel, randomized, placebo-controlled trial, intention to treat population or modified intention to treat population,

Others: crossover trial, non-placebo–controlled trial, nonintention to treat population trial.

**Table 2b.** Sensitivity Analysis of Efficacy of Cholinesterase Inhibitors (Behavioral Disturbance)

Variable	Subgroup	N	n	I^2^	SMD	95% CI	P value	Test for subgroup differences
Blinding	Double blind	12	1793	14	-0.17	-0.27 to -0.06	**0.003**	I^2^ = 98.0 %, **P < 0.00001**
	Others	1	39	NA	-5.02	-6.36 to -3.69	**< 0.00001**	
Cholinesterase inhibitor	Donepezil	9	1130	36	-0.14	-0.31 to 0.02	0.09	I^2^ = 0 %, P = 0.55
	Galantamine	2	82	98	-2.52	-7.35 to 2.31	0.31	
	Rivastigmine	2	620	0	-0.21	-0.37 to -0.04	**0.01**	
Control	Placebo	12	1793	14	-0.17	-0.27 to -0.06	**0.003**	I^2^ = 98.0 %, **P < 0.00001**
	Non-placebo	1	39	NA	-5.02	-6.36 to -3.69	**< 0.00001**	
Diagnosis	DLB	6	500	58	-0.13	-0.40 to 0.14	0.35	I^2^ = 24.2 %, P = 0.27
	PDD + CIPD	6	1289	90	-0.54	-0.98 to -0.10	**0.02**	
	PD	1	43	NA	-0.09	-0.70 to 0.51	0.76	
Duration	12 weeks <	7	1414	88	-0.47	-0.84 to -0.10	**0.01**	I^2^ = 58.9 %, P = 0.12
	12 weeks ≧	6	418	55	-0.1	-0.39 to 0.20	0.52	
Quality of the trial design*	High-quality trials trial design	11	1755	21	-0.17	-0.28 to -0.05	**0.005**	I^2^ = 0 %, P = 0.35
	Others	2	77	98	-2.49	-7.38 to 2.40	0.32	
Sample size	Total n > 28	11	1778	84	-0.3	-0.57 to -0.04	**0.03**	I^2^ = 0 %, P = 0.52
	Total n ≦ 28	2	54	0	-0.11	-0.65 to 0.43	0.69	
Sponsorship	Industry	10	1712	28	-0.17	-0.29 to -0.04	**0.008**	I^2^ = 39.6%, P = 0.20
	Non-industry	3	120	96	-1.62	-3.82 to 0.58	0.15	

CI, confidence interval; CIPD, cognitive impairment in Parkinson’s disease; DLB, Dementia with Lewy bodies; NA, not applicable; PD, Parkinson’s disease; PDD, Parkinson’s disease dementia; SMD, standardized mean difference.

*High-quality trials trial design: double-blind, parallel, randomized, placebo-controlled trial, intention to treat population or modified intention to treat population,

Others: crossover trial, non-placebo–controlled trial, nonintention to treat population trial.

#### Behavioral Disturbance

**Figure 5.**
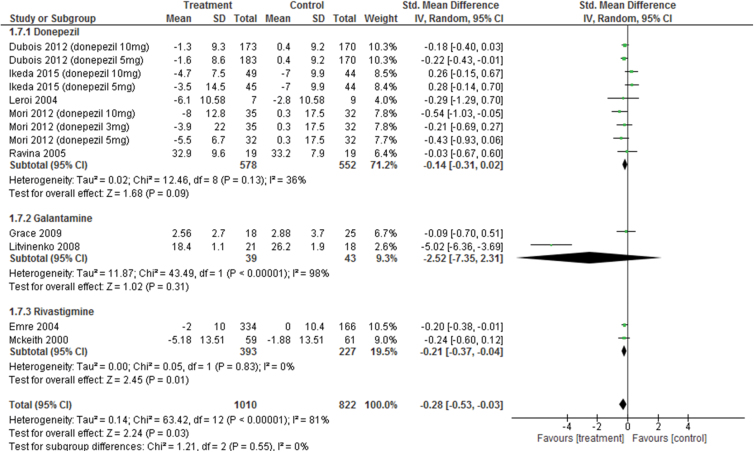
Forest plot of behavioral disturbance (13 comparisons, n = 1832).

Pooled ChEIs improved behavioral disturbance scores compared with control treatments (SMD=−0.28, 95% CI=−0.53 to −0.03, *P*=.03, *I* ^2^=81%, 13 comparisons, n=1832) (Figure 5). Visual inspection of the funnel plots for primary outcomes did not suggest the presence of publication bias (Figure 4b). For individual ChEIs, rivastigmine was significantly more efficacious than placebo (SMD=−0.21, 95% CI=−0.37 to −0.04, *P*=.01, *I* ^2^=0%, 2 comparisons, n=620). There was also a trend toward improved behavioral disturbance scores for donepezil compared with that for placebo (SMD=−0.14, 95% CI=−0.31 to 0.02, *P*=.09, *I* ^2^=36%, 9 comparisons, n=1130), while galantamine had no significant effect compared with control treatment (SMD=−2.52, 95% CI=−7.35 to 2.31, *P*=.31; *I* ^2^=98%, 2 comparisons, n=82).

#### Sensitivity Analyses of Behavioral Disturbance

There was significant heterogeneity in behavioral disturbance scores among the studies (*I* ^*2*^=81%) (Figure 5). Therefore, we performed several sensitivity analyses for identifying confounding factors affecting behavioral disturbance scores (Table 2b). When divided into a double-blind RCT subgroup and “other” subgroup, the significant heterogeneity disappeared in the double-blind RCT subgroup (double-blind RCTs subgroup [n=12], *I* ^2^=14%; other subgroup [n=1 galantamine study (Litvinenko et al., 2008)), *I* ^2^=NA; test for subgroup differences, *I* ^2^=98.0%, *P<*.00001]. When divided into placebo-controlled and non-placebo–controlled trial subgroups, the same results were found. When divided according to the tested ChEIs, the significant heterogeneity disappeared in the donepezil and rivastigmine subgroups (donepezil subgroup: *I* ^2^=36%; rivastigmine subgroup: *I* ^2^=0%); however, heterogeneity remained in the galantamine subgroup (*I* ^2^=98%). When divided into DLB, PDD+CIPD, and PD subgroups, there was significant heterogeneity in the DLB and PDD+CIPD subgroups (DLB: *I* ^2^=58%; PDD+CIPD: *I* ^2^=90%). ChEIs significantly affected behavioral disturbance scores for PDD+CIPD subgroups (SMD=−0.54; 95% CI=−0.98 to −0.10, *P*=.02, *I* ^2^=90%, 6 comparisons, n=1289). In contrast, there was no effect of ChEIs on behavioral disturbance scores in the DLB subgroup (SMD=−0.13, 95% CI=−0.40 to 0.14, *P*=.35, *I* ^2^=58%, 6 comparisons, n=500). We did not perform meta-analysis on behavioral disturbance scores for PD subgroup, because there was only one relevant study (Grace et al., 2009). This study revealed that no significant differences in behavioral disturbance scores were found between groups (SMD=−0.09, 95% CI=−0.70 to 0.51, *P*=.76, *I* ^2^=NA, n=43). When divided into long duration and short duration subgroups, there was significant heterogeneity in both subgroups (long duration subgroup: *I* ^2^=88%; short duration subgroup: *I* ^2^=55%). ChEIs significantly affected behavioral disturbance scores for long duration subgroups (SMD=−0.47; 95% CI=−0.84 to −0.10, *P*=.01, *I* ^2^=88%, 7 comparisons, n=1414). In contrast, there was no effect of ChEIs on behavioral disturbance scores in the short duration subgroups (SMD=−0.1, 95% CI=−0.39 to 0.20, *P*=.52, *I* ^2^=55%, 6 comparisons, n=418). When divided into high-quality trial and other subgroups, the significant heterogeneity disappeared in the high-quality trials subgroup (*I* ^2^=21%) but remained in the “other” subgroup (*I* ^2^=98). ChEIs significantly affected behavioral disturbance scores for high-quality trial subgroups (SMD=−0.17; 95% CI=−0.28 to −0.05, *P*=.005, *I* ^2^=21%, 11 comparisons, n=1755). In contrast, there was no effect of ChEIs on behavioral disturbance scores in the other subgroups (SMD=−2.49, 95% CI=−7.38 to 2.40, *P*=.32, *I* ^2^=98%, 2 comparisons, n=77). Further, when divided into large and small sample size subgroups, the significant heterogeneity disappeared in the small sample size subgroup (*I* ^2^=0%) but remained in the large sample size subgroup (*I* ^2^=84%). ChEIs significantly affected behavioral disturbance scores for large sample size subgroup (SMD=−0.3; 95% CI=−0.57 to −0.04, *P*=.03, *I* ^2^=84%, 11 comparisons, n=1778). In contrast, there was no effect of ChEIs on behavioral disturbance scores in the small sample size subgroup (SMD=−0.11, 95% CI=−0.65 to 0.43, *P*=.69, *I* ^2^=0%, 2 comparisons, n=54). When divided into industry and nonindustry subgroups, the significant heterogeneity disappeared in the industry subgroup (*I* ^2^=28%) but remained in the nonindustry subgroup (*I* ^2^=96%). ChEIs significantly affected behavioral disturbance scores for industry subgroup (SMD=−0.17; 95% CI=−0.29 to −0.04, *P*=.008, *I* ^2^=28%, 10 comparisons, n=1712). In contrast, there was no effect of ChEIs on behavioral disturbance scores in the nonindustry subgroup (SMD=−1.62, 95% CI=−3.82 to 0.58, *P*=.15, *I* ^2^=96%, 3 comparisons, n=120).

#### Motor Function

**Figure 6.**
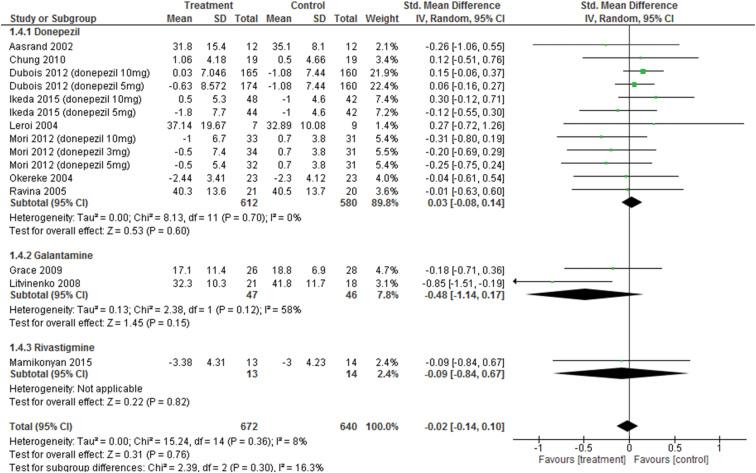
Forest plot of motor function (15 comparisons, n = 1312).

Changes in UPDRS-motor scores were not significantly different from control treatments (SMD=−0.02, 95% CI=−0.14 to 0.10, *P*=.76, *I* ^2^=8%, 15 comparisons, n=1312) (Figure 6). Visual inspection of the funnel plots for primary outcomes did not suggest the presence of publication bias (Figure 4c). For individual ChEIs (donepezil, galantamine, and rivastigmine), no significant differences in UPDRS-motor scores were found between groups.

### Meta-Analysis for Secondary Outcomes

#### Activities of Daily Living

Pooled ChEIs improved ADL scores compared with placebo (SMD=−0.28) (Table 3). For individual ChEIs, donepezil significantly improved ADL scores compared with placebo (SMD=−0.37). We could not perform a meta-analysis for rivastigmine, because there was only one study reporting ADL scores (Emre et al., 2004); however, this study revealed that rivastigmine significantly improved ADL scores compared with placebo (SMD=−0.21).

**Table 3.** Meta-Analysis of Secondary Outcomes of Cholinesterase Inhibitors

Outcome	ChEI	N	n	I^2^	SMD	95% CI	P		
Activities of daily living	Donepezil	6	373	0	-0.37	-0.58 to -0.17	**0.0004**		
	Galantamine	0	0	NA	NA	NA	NA		
	Rivastigmine	1	498	NA	-0.21	-0.40 to -0.02	**0.003**		
	Pooled ChEIs	7	871	0	-0.28	-0.42 to -0.15	**<0.0001**		
Global function assessment	Donepezil	8	968	71	-0.61	-0.89 to -0.33	**<0.0001**		
	Galantamine	0	0	NA	NA	NA	NA		
	Rivastigmine	2	521	0	-0.34	-0.53 to -0.16	**0.0002**		
	Pooled ChEIs	10	1489	62	-0.52	-0.73 to -0.31	**<0.00001**		
Outcome	ChEI	N	n	I^2^	RR	95% CI	P for RR	NNH	P for NNH
Discontinuation rate due to all causes	Donepezil	8	925	0	1.33	0.99 to 1.78	0.06		
	Galantamine	2	110	65	1.04	0.09 to 12.57	0.98		
	Rivastigmine	5	731	0	1.59	1.16 to 2.17	**0.004**	NS	
	Pooled ChEIs	15	1766	0	1.48	1.20 to 1.82	**0.0002**	14	0.02
Discontinuation rate due to adverse events	Donepezil	8	925	0	1.35	0.92 to 1.97	0.13		
	Galantamine	2	110	NA	2.45	1.00 to 5.98	0.05		
	Rivastigmine	4	703	14	1.7	0.94 to 3.08	0.08		
	Pooled ChEIs	14	1738	0	1.59	1.20 to 2.10	**0.001**	20	0.04
At least one adverse events	Donepezil	7	964	0	1.08	0.99 to 1.18	0.1		
	Galantamine	1	69	NA	1.12	0.97 to 1.29	0.13		
	Rivastigmine	3	691	6	1.18	1.08 to 1.30	**0.0005**	9	0.04
	Pooled ChEIs	11	1724	0	1.13	1.06 to 1.19	**<0.0001**	11	0.0001
Severe adverse events	Donepezil	3	831	32	0.97	0.45 to 2.10	0.95		
	Galantamine	0	0	NA	NA	NA	NA		
	Rivastigmine	2	150	0	1.16	0.51 to 2.66	0.72		
	Pooled ChEIs	5	981	0	1.21	0.83 to 1.76	0.31		
Outcome	ChEI	N	n	I^2^	RR	95% CI	P for RR	NNH	P for NNH
Diarrhea	Donepezil	5	806	19	1.2	0.55 to 2.60	0.65		
	Galantamine	0	0	NA	NA	NA	NA		
	Rivastigmine	1	541	NA	1.61	0.74 to 3.48	0.23		
	Pooled ChEIs	6	1347	3	1.34	0.81 to 2.24	0.26		
Dizziness	Donepezil	4	768	32	1.27	0.77 to 2.10	0.35		
	Galantamine	0	0	NA	NA	NA	NA		
	Rivastigmine	1	541	NA	5.19	1.23 to 21.90	**0.02**	20	**0.001**
	Pooled ChEIs	5	1309	66	1.81	0.85 to 3.85	0.12		
Hallucination	Donepezil	2	689	0	0.6	0.31 to 1.17	0.13		
	Galantamine	0	0	NA	NA	NA	NA		
	Rivastigmine	2	597	29	0.64	0.26 to 1.62	0.35		
	Pooled ChEIs	4	1286	0	0.58	0.37 to 0.91	**0.02**	NS	
Insomnia	Donepezil	3	626	60	1.33	0.22 to 8.00	0.76		
	Galantamine	0	0	NA	NA	NA	NA		
	Rivastigmine	1	56	NA	2	0.40 to 10.05	0.4		
	Pooled ChEIs	4	682	40	1.66	0.51 to 5.39	0.4		
Nausea	Donepezil	6	948	0	2.39	1.46 to 3.90	**0.0005**	NS	
	Galantamine	0	0	NA	NA	NA	NA		
	Rivastigmine	1	541	NA	2.6	1.67 to 4.04	**<0.0001**	6	**<0.00001**
	Pooled ChEIs	7	1489	0	2.5	1.80 to 3.47	**<0.00001**	13	**0.05**
Parkinson symptoms	Donepezil	3	831	0	1.58	0.91to 2.75	0.11		
	Galantamine	1	69	NA	1.36	0.80 to 2.32	0.26		
	Rivastigmine	1	30	NA	0.29	0.01 to 6.69	0.44		
	Pooled ChEIs	5	930	0	1.43	0.97 to 2.09	0.07		
Outcome	ChEI	N	n	I^2^	RR	95% CI	P for RR	NNH	P for NNH
Tremor	Donepezil	1	550	NA	2.48	0.97 to 6.33	0.06		
	Galantamine	1	69	NA	2.04	0.71 to 5.88	0.19		
	Rivastigmine	2	597	0	2.33	1.18 to 4.58	**0.01**	17	**0.002**
	Pooled ChEIs	4	1216	0	2.3	1.41 to 3.75	**0.0008**	20	**<0.0001**
Vomiting	Donepezil	2	689	71	1.73	0.10 to 29.06	0.7		
	Galantamine	0	0	NA	NA	NA	NA		
	Rivastigmine	1	541	NA	9.89	3.15 to 31.10	**<0.0001**	7	**<0.00001**
	Pooled ChEIs	3	1230	61	4.09	0.90 to 18.67	0.07		

ChEI, cholinesterase inhibitor; CI, confidence interval; N, number of comparisons; n, number of patients; NA, not applicable; NNH, number needed to harm; NS, not significant; RR, risk ratio; SMD, standardized mean difference.

#### Global Function Assessment

Pooled ChEIs improved global function assessment scores compared with placebo (SMD=−0.52) (Table 3). For individual ChEIs, donepezil and rivastigmine significantly improved global function assessment scores compared with placebo (donepezil: SMD=−0.61 and rivastigmine: SMD=−0.34).

#### Safety Outcomes

There was a significantly higher all-cause discontinuation rate in the pooled ChEIs group compared with controls (RR=1.48, NNH=14) (Table 3). Rivastigmine was associated with higher all-cause discontinuation rate than placebo (RR=1.59, NNH was not significant).

There was a significantly higher discontinuation rate because of adverse events in the pooled ChEIs group compared with the control group (RR=1.59, NNH=20) (Table 3).

There was a significantly higher rate of at least one adverse event between pooled ChEIs and placebo groups (RR=1.13, NNH=11) (Table 3). For individual ChEIs, there was a significantly higher rate of at least one adverse event for rivastigmine compared with placebo (RR=1.18, NNH=9). For donepezil and galantamine, the rates of at least one adverse event were similar between groups. The incidence of severe adverse events was similar between pooled ChEIs and placebo groups. The rates of severe adverse events were similar between groups (there were no data for galantamine).

With respect to individual adverse events, the pooled ChEIs group treatment was associated with a lower incidence of hallucination than placebo group (RR=0.58, NNH was not significant) (Table 3). The pooled ChEIs group had a higher incidence of nausea than the placebo group (RR=2.50, NNH=13). For individual ChEIs, donepezil was associated with a higher incidence of nausea than placebo (RR=2.39, NNH was not significant). Rivastigmine was associated with a higher incidence of nausea than placebo (RR=2.60, NNH=6). The pooled ChEIs group had a higher incidence of tremor than the placebo group (RR=2.30, NNH=20). For individual ChEIs, rivastigmine was associated with a higher incidence of tremor than placebo (RR=2.33, NNH=17). No significant differences were found in the incidences of diarrhea, vomiting, PD symptoms, insomnia, and dizziness between pooled ChEIs and placebo groups. Rivastigmine was associated with a higher incidence of vomiting and dizziness than placebo (vomiting: RR=9.89, NNH=7; dizziness: RR=5.19, NNH=20).

## Discussion

To our knowledge, this is the first comprehensive meta-analysis of RCTs assessing the efficacy and safety of ChEIs for Lewy body disorders. The main results indicate that ChEIs improve cognitive function, behavioral disturbances, ADL, and global function compared with placebo. Moreover, pooled ChEIs did not worsen motor function. According to the effect sizes of individual ChEIs for cognitive function, donepezil was the most effective (SMD=−0.51), followed by rivastigmine (SMD=−0.29), while galantamine had no significant effect compared with placebo. Further, only rivastigmine significantly improved behavioral disturbances compared with placebo; however, even these effects were small (SMD=−0.21). We suggest that the significant heterogeneity among studies of cognitive function and behavioral disturbances was because of variation in quality of the trial design, because the heterogeneity was reduced after excluding one open-label, non-placebo–controlled trial (Litvinenko et al., 2008). Sensitivity analysis also revealed heterogeneity because of blinding methods. One previous meta-analysis of DLB, PDD, and CIPD (Wang et al., 2015) reported that ChEIs were superior to placebo in several efficacy outcomes (cognitive function, behavioral disturbances, ADL, global function, and motor function) similar to the results of our meta-analysis. Further, we found that ChEIs significantly improved cognitive function in the DLB and PDD + CIPD patient groups, but not in the PD group. These results are strongly suggesting considerations for approving ChEIs for Lewy Body disorders to treat cognitive decline. In addition, we found that ChEIs significantly improved behavioral disturbances in PDD+CIPD patient group, but not in the DLB and PD groups. Further, we found that ChEIs significantly improved ADL in patients with DLB and PDD+CIPD. Moreover, because number of studies of galantamine and rivastigmine were small, a multiple-network meta-analysis of all anti-dementia drugs including memantine will be required to increase a statistical power.

While these drugs were effective against several cardinal deficits associated with Lewy body diseases, there were also significantly higher rates of all-cause discontinuation, discontinuation because of adverse events, and incidence of at least one adverse event in the pooled ChEIs group compared with controls. Moreover, ChEI treatment was associated with a higher incidence of nausea and tremor compared with placebo. For individual ChEIs, rivastigmine was associated with a higher incidence of vomiting and dizziness compared with placebo.

These conclusions must be considered considering several limitations. The first limitation is that our meta-analysis includes “gray” studies supported by pharmaceutical companies. However, these represented the majority of the retrieved articles. Nonetheless, they appear in peer-reviewed journals. Moreover, there were no significant subgroup differences between industry-sponsored and non-industry–sponsored studies (*I* ^*2*^=39.6%, *P*=.20). The second limitation is that characteristics the studies included in the meta-analysis (Table 1). The third limitation is that included studies had several risk of bias (Figure 2). The fourth limitation is that patients with dementia are known to have a poor drug compliance (Boada and Arranz, 2013), reducing the measured effectiveness. Finally, several studies included in this meta-analysis did not report any available data on symptom scales and safety outcomes; therefore, the outcome results for efficacy and safety did not include data from all the 17 studies.

In conclusion, ChEIs are beneficial for the treatment of Lewy body disorders as assessed by multiple scales evaluating cognition, behavioral disturbances, ADL, and global function. Moreover, ChEIs do not worsen motor function. However, a careful monitoring of treatment compliance and side effects is required.

## Statement of Interest

Dr. Matsunaga has received speaker’s honoraria from Eisai, Janssen, Novartis, Daiichi Sankyo, Ono, Eli Lilly, Takeda, and Otsuka. Dr. Kishi has received speaker’s honoraria from Abbott, Astellas, Daiichi Sankyo, Dainippon Sumitomo, Eisai, Eli Lilly, GlaxoSmithKline, Yoshitomi, Otsuka, Meiji, Shionogi, Janssen, Novartis, Tanabe-Mitsubishi, and Pfizer. Dr. Yasue has received speaker’s honoraria from Otsuka, Eli Lilly, Janssen, Novartis, and Eisai. Dr. Iwata has received speaker’s honoraria from Astellas, Dainippon Sumitomo, Eli Lilly, GlaxoSmithKline, Janssen, Yoshitomi, Otsuka, Meiji, Shionogi, Novartis, and Pfizer.

All authors declare that they have no direct conflicts of interest relevant to this study. No grants or other funding sources were used for this study.
